# Sphingolipids in High Fat Diet and Obesity-Related Diseases

**DOI:** 10.1155/2015/520618

**Published:** 2015-11-16

**Authors:** Songhwa Choi, Ashley J. Snider

**Affiliations:** ^1^Department of Medicine and Molecular and Cellular Biology, Stony Brook University, Stony Brook, NY 11794, USA; ^2^Stony Brook Cancer Center, Stony Brook University, Stony Brook, NY 11794, USA; ^3^Northport VA Medical Center, Northport, NY 11768, USA

## Abstract

Nutrient oversupply associated with a high fat diet (HFD) significantly alters cellular metabolism, and specifically including sphingolipid metabolism. Sphingolipids are emerging as bioactive lipids that play key roles in regulating functions, in addition to their traditional roles as membrane structure. HFD enhances *de novo* sphingolipid synthesis and turnover of sphingolipids via the salvage pathway, resulting in the generation of ceramide, and more specifically long chain ceramide species. Additionally, HFD elevates sphingomyelin and sphingosine-1 phosphate (S1P) levels in several tissues including liver, skeletal muscle, adipose tissue, and cardiovascular tissues. HFD-stimulated sphingolipid generation contributes to systemic insulin resistance, dysregulated lipid accumulation, and cytokine expression and secretion from skeletal muscle and adipose tissues, exacerbating obesity-related conditions. Furthermore, altered sphingolipid levels, particularly ceramide and sphingomyelin, are involved in obesity-induced endothelial dysfunction and atherosclerosis. In this review, HFD-mediated sphingolipid metabolism and its impact on HFD-induced biology and pathobiology will be discussed.

## 1. Introduction

Obesity is emerging as a significant public health concern in developed countries. Obesity contributes significantly to increasing medical costs by exacerbating other chronic diseases, such as cardiovascular disease, type 2 diabetes, and certain types of cancers. In the United States, more than one-third (34.9%) of adults and 17% of the youth are obese [1]. A western diet comprised of a high fat content is a primary contributing factor for obesity which has led to extensive studies using models of high fat diet (HFD) to delve into the pathobiologies of obesity-related diseases.

Sphingolipids are bioactive lipids which are involved in cellular signaling and regulatory functions [2]. These lipid species have been implicated as potent mediators in the regulation of several diseases such as cancer and inflammatory diseases [2, 3]. Based on these studies, interest for the role of sphingolipids in obesity-induced pathobiology is emerging. This is of particular interest as HFD significantly changes energy metabolism, including sphingolipid levels, suggesting the possibility that HFD-induced dysregulation of sphingolipid metabolism contributes to HFD and obesity-related pathologies. This review will discuss sphingolipid metabolism altered by HFD and its impact on HFD-induced biology and pathobiology.

## 2. Sphingolipid Metabolism

Sphingolipid metabolism is a complex network composed of numerous metabolizing enzymes that function to generate sphingolipids through either* de novo* synthesis or the salvage pathway. Sphingolipid levels are tightly regulated by these enzymes, allowing them to function as bioactive mediators.* De novo* sphingolipid synthesis begins with the enzyme serine palmitoyltransferase (SPT). SPT functions as a heterodimer with subunits SPTLC1 and SPTLC2, or SPTLC3, primarily using palmitoyl-CoA as a substrate. SPTLC3 has recently been shown to utilize myristoyl-CoA as well [4]. Condensation of serine and palmitoyl-CoA (or myristoyl-CoA) which forms 3-ketosphinganine is then reduced to form dihydrosphingosine by NADH-dependent reductase. Ceramide synthases (CerS), of which there are six, catalyze the acylation of dihydrosphingosine to dihydroceramide. Subsequent desaturation yields the generation of ceramide from dihydroceramide. Ceramide can also be generated by sphingomyelinases (SMase) and glucosylceramidase (GCase) from various membrane glycolipids and sphingolipids in the salvage pathway. Once formed, ceramide acts as a central hub in the sphingolipid network. Ceramide can be phosphorylated by ceramide kinase to form ceramide 1-phosphate (C1P). Sphingomyelin synthase (SMS) and glucosyl- or galactosyl-ceramide synthases (GCS) incorporate ceramide into sphingomyelin and glycosyl or galactosylceramide, respectively. Finally, one of five ceramidases (CDase) facilitates the deacylation of ceramide to produce sphingosine, followed by conversion to sphingosine-1 phosphate (S1P) by sphingosine kinases (SKs) or reacylation to ceramide by CerS. S1P can be degraded by one of two enzymes, sphingosine phosphate phosphatase (SPP) or S1P lyase. SPP dephosphorylates S1P to sphingosine, allowing for the reformation of ceramide, whereas S1P lyase irreversibly breaks down S1P to ethanolamine phosphate and hexadecanal, resulting in exit from sphingolipid metabolism.

## 3. The Effects of HFD on Sphingolipid Metabolism

### 3.1. Ceramide

Dysregulation of ceramide in response to nutrient oversupply, specifically saturated fatty acids, has been known to be a key factor in the impairment of cellular homeostasis and function [5]. During* de novo* synthesis, one of the six isoforms of CerS generates ceramide species with specific fatty acid chain lengths [6]. The long chain fatty acid palmitate (palmitoyl-CoA) is the main source of fatty acid in* de novo* sphingolipid synthesis. HFD administration and/or palmitate treatment have been shown to increase ceramide content independent of tissue or cell type (Table 1). Several tissues including liver, adipose, skeletal muscle, and heart demonstrate elevated total ceramide and long chain ceramide levels upon HFD administration. Diabetic models using HFD have also shown elevations in circulating ceramides [7]. Additionally, several studies have demonstrated C16:0 and C18:0 ceramides are consistently elevated by HFD/palmitate [8–13], compared to ceramides with the other chain lengths. Interestingly, very long chain ceramides, C24:0 and C24:1, have been shown to significantly increase upon HFD/palmitate treatment in some studies [8, 14–16] but are decreased in others [9, 11, 17] (Table 1). Additionally, ceramide induction in response to HFD has also been demonstrated as an aging process with some studies indicating augmented accumulation of ceramide in muscle in aging individuals [18–21]. The mechanism for this age-related increase in ceramide is still unclear, but the membrane protein CD36/FAT that facilitates the uptake of fatty acid has been suggested as a potential mechanism in HFD administration [20].

HFD-induced ceramide generation is primarily due to increased* de novo* synthesis, whereby excessive fatty acid supply supplies a continuous substrate supply for this pathway. Radioactive palmitate treatment in HepG2 cells (human hepatocarcinoma cell line) and C2C12 cells (murine myotubes) increased incorporation of palmitate into ceramide, demonstrating that fatty acids from exogenous sources can be utilized for sphingolipid synthesis [14, 22]. In addition to increasing substrate, HFD/palmitate alters expression and activity of the enzymes involved in* de novo* synthesis. HFD increased transcription of SPT subunits, SPTLC1, SPTLC2, and SPTLC3 [9, 18, 23–25], as well as activity of SPT [26]. CerS have also been shown to be upregulated by HFD [9, 17, 24, 25], specifically CerS1 and CerS6, which are key in the formation of long chain ceramide species. This upregulation resulted in the generation of C16:0 ceramide in response to HFD [6, 17]. In addition to CerS1 and CerS6, CerS2 and CerS4 expression has been reported to increase in response to HFD/palmitate [23, 24].

Upstream regulation of* de novo* sphingolipid synthesis by HFD has not been well studied but there are suggestions in the literature. TLR4, suggested as a receptor for saturated fatty acids [27], has also been shown to mediate several HFD-induced cell signaling events including HFD-induced* de novo* sphingolipid synthesis [28]. TLR4 deletion in skeletal muscle inhibited increases in ceramide levels upon HFD administration. Additionally, silencing the downstream signaling mechanism NF-*κ*B suppressed induction of mRNA expression in* de novo* sphingolipid enzymes, such as SPTLC1, SPTLC2, CerS1, CerS2, CerS5, and CerS6. Cannabinoid-1 receptor (CB_1_R) is another potential regulatory mechanism for* de novo* sphingolipid synthesis, as hepatic CB_1_R deletion or blockade suppressed HFD-induced SPTLC3, CerS1, and CerS6 upregulation and subsequent ceramide increases [9]. Additionally, the energy sensing molecule, AMP-activated protein (AMPK), has been shown to regulate* de novo* sphingolipid synthesis. The AMPK activator, AICAR, suppressed HFD-induced transcription of SPTLC2 in skeletal muscle, suggesting AMPK as an upstream regulator of SPTLC2 [23]. Adipocyte-derived plasminogen activator inhibitor-1 (PAI-1) is important in the regulation of ceramide synthesis in adipose tissue as well [16]. PAI-1 deficient mice exhibited decreased expression of SPTLC2, SPTLC3, and CerS1 upon HFD feeding, resulting in reduced adipose ceramide level [16].

Enzymes of the salvage pathway have also been implicated in dietary manipulations of ceramide levels. Radio-labeled palmitate has been used to demonstrate the involvement of CerS and not* de novo* sphingolipid synthesis, as labeled palmitate was found in the acyl chain of ceramide, and not in the sphingoid backbone [14]. In addition to CerS, HFD administration enhanced mRNA expression and activity of acid SMase (aSMase) and neutral SMase (nSMase) in liver [24, 29] and adipose tissue [16]. Pharmacological inhibition of aSMase, using amitriptyline, inhibited ceramide induction by HFD in plasma and adipose tissue [30]. However, compared to studies examining* de novo* sphingolipid synthesis, regulation of the salvage pathway has not been well-studied.

Taken altogether, the above studies demonstrate that HFD and palmitate regulate global sphingolipid metabolism through both* de novo* sphingolipid synthesis and the salvage pathway. These diet-induced alterations in ceramide demonstrate that nutrition has the ability to alter sphingolipid metabolism and in turn downstream signaling pathways to induce obesity-related conditions, such as insulin resistance and ectopic lipid accumulation.

### 3.2. Sphingomyelin

Sphingomyelin, one of the most abundant sphingolipid species, plays key roles in membranes and has been suggested to function in lipid rafts [2, 31]. Concentrations of sphingomyelin are higher than ceramide, suggesting that sphingomyelin may act as a pool for the rapid generation of ceramide. It has been demonstrated that high concentrations of serum sphingomyelin correlate with coronary artery disease in obese individuals [32]; and several studies demonstrated that HFD or palmitate administration increased sphingomyelin levels in cells and tissues, including serum (Table 2) (although the magnitude of sphingomyelin increase was smaller than that of ceramide). Similar to alterations in ceramide species, long chain sphingomyelin species, mostly C16:0 and C18:0, have been reported to increase by HFD or palmitate treatment in liver, adipose tissue, and plasma [11, 29, 33] (Table 2). Macrophages stimulated with palmitate demonstrated increases in specific species of sphingomyelin, specifically 18:0, 20:0, 22:0, and 22:1 [34]. HFD-induced changes in sphingomyelin have been attributed to SMS2 activity and expression. Mice overexpressing liver-specific SMS2 exhibited elevated HFD-induced sphingomyelin in plasma. Additionally, this study demonstrated loss of SMS2* in vivo* repressed HFD-induced increases in sphingomyelin [35].

The role for sphingomyelin in HFD-induced biologies has been less studied than ceramide; however, sphingomyelin levels are elevated in diverse tissues, including liver, skeletal muscle, adipose, and cardiac, as well as plasma. These studies suggest that sphingomyelin may directly regulate cell functions and/or serve as a reservoir for the generation of ceramide.

### 3.3. Sphingosine/S1P

Sphingosine, the breakdown product of ceramide, is known to regulate apoptosis and cell-cycle arrest [36–38]. There are few studies examining the role of sphingosine in HFD-mediated processes. It is possible that sphingosine is rapidly converted to S1P (or reacylated to ceramide) in HFD-mediated sphingolipid metabolism. However, the exact role of sphingosine in HFD-mediated effects has yet to be determined. S1P, a potent bioactive sphingolipid, is present in low nanomolar concentrations in the cell and has high affinity to S1PRs functioning as a signaling molecule. Despite its potent capacity as a bioactive lipid, studies for S1P (and its precursor, sphingosine) upon HFD administration are limited (compared to those examining ceramide). However, S1P (and sphingosine) levels exhibited significant increases in liver, skeletal muscle, and plasma in response to several experimental models of HFD. Conversely, there are also a few opposing studies that have demonstrated decreases in the levels of these lipids (Table 3). While S1P in liver and skeletal muscle was unchanged by HFD or palmitate in some studies [39–41], S1P in plasma was consistently elevated, which suggests that HFD-enhanced S1P levels in circulation may mediate some of the systemic effects associated with HFD and obesity. Furthermore, elevated plasma S1P positively correlated with body fat percentage, body mass index, waist circumference, and fasting plasma insulin in obese humans [42]. In cell culture palmitate stimulation of mouse derived pancreatic beta cells, MIN6, and primary rat hepatocytes resulted in increased S1P secretion [43, 44].

HFD-induced S1P seems to be due to augmented expression and activity of SK. Two isoforms for mammalian SK have been identified, SK1 and SK2. These two isoforms have suggested differences in subcellular localization, cytosol for SK1 and nucleus for SK2, as well as different physiological functions [45–47]. With respect to HFD, both enzymes have been shown to mediate HFD-induced S1P; however, regulation of these enzymes is suggested to be different. Skeletal muscle from mice fed a HFD for 16 weeks exhibited increases in only SK1 mRNA expression, and not SK2 [48]. In contrast, liver from rats fed a HFD for two weeks exhibited increased expression and activity of SK2, rather than SK1 [49].

The studies examining the effects of HFD on sphingosine and S1P levels suggest that S1P levels are elevated in several tissues. Moreover, S1P levels in plasma were elevated in response to HFD and have been suggested to be secreted from tissues including the liver and pancreas. This S1P has been shown to be generated by both isoforms of SK which are altered by HFD; however, they seem to be independently regulated each by independent mechanisms that have yet to be fully elucidated.

## 4. Sphingolipids in HFD-Induced Pathobiology

Obesity is well-known to induce several pathologic conditions, including insulin resistance. Insulin is responsible for clearance of redundant nutrients in circulation by facilitating their uptake and storage in liver, skeletal muscle, and adipose tissue. Under normal physiologic conditions, increased blood glucose from the diet stimulates insulin secretion and subsequent reduction of blood glucose, working as a negative feedback mechanism. However, when insulin fails to clear glucose in bloodstream this results in “insulin resistance.” At the molecular level, insulin triggers signaling through the insulin receptor and subsequent phosphorylation of the insulin receptor substrate (IRS) [50]. Phosphorylated IRS recruits phosphatidylinositol 3-kinase (PI3K), which results in phosphorylation of Akt and its various substrates, including glycogen synthase kinase 3*β* (GSK3*β*) regulating glucose metabolism and glycogen, lipid, and protein synthesis [50, 51]. In insulin resistance, this signaling pathway is disrupted and ceramide has been reported to play a key role in the processes involved in insulin resistance [5].

Disruption of various enzymes in sphingolipid metabolism including SPT, CerS6, aSMase, and SMS2 suppressed weight gain by HFD [17, 30, 52, 53]. Specifically, myriocin, an inhibitor of* de novo* sphingolipid synthesis, enhanced energy expenditure and improved leptin resistance associated with HFD inhibiting obesity [52]. Additionally, for decades the role for sphingolipids in postobesity events such as ectopic lipid accumulation and insulin resistance has been established. Induction of ceramide by palmitate has been shown to disturb insulin signaling via inhibition of Akt signaling mediated by TLR4-NF-*κ*B in myotubes [28]. Many studies demonstrated that inhibition of* de novo* synthesis of sphingolipids suppressed blood glucose and insulin induction [17, 40, 52, 54]. In addition to these systemic effects, aberrant sphingolipid regulation plays a significant role in HFD-induced dysregulation of various cellular signaling pathways. Understanding the specific effects in specific tissues will begin to lend insight into sphingolipid metabolism as a therapeutic target for HFD-induced pathologic conditions.

### 4.1. Liver

The liver is the central organ for the regulation of metabolism by action of insulin. Obesity-induced hepatic insulin resistance results in expansion of peripheral insulin resistance due to elevated fasting blood glucose and subsequent blood insulin levels. Sphingolipids play a significant role in energy metabolism; therefore, there has been an effort to elucidate the role for sphingolipids, particularly, ceramide and S1P, in hepatic insulin resistance.

Increased hepatic ceramide has been suggested to be a major mechanism in the regulation of insulin resistance, and suppression of ceramide induction has been demonstrated to improve insulin signaling. Direct treatment of Hu7 hepatoma cells with short chain C6:0 ceramide decreased phosphorylation of Akt and GSK3*β* [24]. The CB_1_R agonist anandamide increased endogenous C16:0 ceramide in hepatocytes via* de novo* synthesis, resulting in inhibition of insulin receptor substrate 1 (IRS1) phosphorylation, increased Akt phosphatase Phlpp1 expression, and suppression of Akt signaling in response to insulin [9]. Moreover, inhibition of ceramide synthesis through pharmacological inhibitors and gene deletion improved insulin signaling. Myriocin treatment and CerS6 deletion restored insulin-induced Akt signaling previously disrupted by ceramide [9, 17]. In contrast, CerS2 haploinsufficiency reduced very long chain ceramide and inhibited insulin sensitivity. This contradictory result was suggested to be due to CerS6 upregulation and subsequent C16:0 ceramide synthesis upon HFD [55]. These studies suggest that the roles for ceramide in energy metabolism regulation may be species-dependent and that many enzymes may generate ceramide in the liver during insulin resistance.

Hepatic S1P and its receptors are also suggested to function as key molecules in the regulation of insulin signaling. Palmitate-induced production and secretion of S1P from hepatocytes led to not only abrogation of insulin-induced Akt activation, but also reduced glucokinase expression and consequent glycogen synthesis [43]. These signaling pathways are reversed by SKII and JTE-013 treatment, an SK1 inhibitor, and an S1P receptor 2 (S1PR2) antagonist, respectively [43]. SK2 overexpression enhanced insulin sensitivity in liver from HFD-fed mice [56], implicating localization of S1P may be important for regulation of hepatic insulin resistance.

In addition to ceramide and S1P, sphingomyelin also may be involved in the regulation of insulin sensitivity. SPTLC2 or SMS2 deletion and subsequent decreases in sphingomyelin levels in the plasma membrane restored insulin signaling disrupted by HFD. Also exogenous sphingomyelin treatment impaired insulin-stimulated Akt phosphorylation [54].

Nonalcoholic steatohepatitis (NASH) is a major obesity-related disease that involves elevated proinflammatory cytokine activation, oxidative stress with mitochondrial dysfunction, leading to fibrogenesis, and finally liver cirrhosis [57–59]. The progress of these diseases is closely associated with dysregulated intracellular lipid accumulation and metabolism [57], and recently the involvement of sphingolipids in this process has been demonstrated. Inhibition of* de novo* sphingolipid synthesis with myriocin reduced triglyceride (TG) accumulation in liver [40]. Similarly, CerS6 deletion reduced CD36/FAT expression and increased palmitate *β*-oxidation, resulting in suppressed lipid accumulation [17]. Increased ceramide breakdown by aCDase induction prevented TG accumulation in liver from HFD feeding [60]. It has also been well documented that obesity induces ER stress [61] and important mechanism in the activation of lipogenesis [62]. Indeed, inhibition of the salvage pathway via aSMase deletion ameliorated HFD-induced hepatic steatosis via protection from ER stress [63]. However, acute ER stress augmented SK2 expression, inhibiting HFD-induced lipid accumulation in the liver [56], potentially functioning as an initial defense mechanism against HFD-induced lipotoxicity. In addition to the study demonstrating overexpression of SK2 suppressed lipid accumulation [56], deletion of SK2 significantly intensified HFD-induced hepatic steatosis [49]. The latter study demonstrated nuclear S1P was critical for histone acetylation and followed global gene transcription in lipid metabolism [49]. These studies highlight the importance of subcellular localization of sphingolipid metabolizing enzymes and their lipid products suggesting that specific pools of sphingolipids function to differentially regulate hepatic insulin resistance.

These studies demonstrate that HFD-induced alteration in sphingolipid metabolism disrupts normal physiology in liver. Specifically, increases in ceramides have been demonstrated to play a key role in insulin resistance, while future studies will be needed to determine the specific roles of sphingomyelin, sphingosine, and S1P.

### 4.2. Skeletal Muscle

Skeletal muscle is a primary site for glucose utilization and is significantly affected by HFD. Similar to the liver, HFD induces insulin resistance in skeletal muscle, inhibiting glucose uptake [64]. In myotubes palmitate-induced ceramide generation and exogenous C2-ceramide treatment impaired insulin-stimulated Akt activation [65]. Additionally, ceramide-induced PKC*ζ* activation triggered the inhibitory phosphorylation of Akt [66]. Inhibition of nSMase, which should decrease ceramide generation, abolished palmitate-induced JNK and NF-*κ*B activation and ER stress, conferring improvement in insulin resistance [67]. Furthermore, ceramide has also been implicated in the regulation of ectopic lipid accumulation in skeletal muscle and aggravation of insulin resistance [21, 68].

Recently, skeletal muscle has emerged as secretory tissue for cytokines, termed myokines [69]. Interleukin-6 (IL-6), the first described myokine, was found to be released from contracting skeletal muscle [70]. In relation to HFD, in C2C12 and mouse primary myotubes, palmitate stimulated IL-6 expression and secretion in an SK1- and S1PR3-dependent manner [48]. These data suggest that SK1, S1P, and S1PRs may play a role in skeletal muscle myokine generation. However, the definitive role for IL-6 from skeletal muscle is controversial in HFD-induced metabolic disorders [64].

### 4.3. Adipose Tissue

The traditional perception of adipose tissue is that of long-term energy storage. However, since the discovery of increased of tumor necrosis factor alpha (TNF*α*) in adipose tissue from obese individuals [71], this tissue has become the focus of numerous studies on the secretion of proinflammatory cytokines and consequent pathobiologies including insulin resistance [72, 73]. After identification of TNF*α*, various cytokines including leptin, PAI-1, and adiponectin have been defined as adipokines [74–76]. Adipose tissue consists primarily of adipocytes with minor population of preadipocytes and immune cells, such as lymphocytes and macrophages. Infiltration of macrophages occurs via recruitment by chemokines and cytokines secreted by adipose tissue resulting in exacerbation of obesity-induced proinflammatory responses [73]. HFD-induced ceramide levels have also been implicated in adipokine induction and secretion. CerS6 deletion, and subsequent decreased ceramide levels, inhibited expression of proinflammatory cytokines including IL-6 in response to HFD [17]. In addition,* de novo* synthesis of ceramide contributed to HFD stimulation of monocyte chemoattractant protein-1 (MCP-1), suggesting that ceramide may play a role in recruitment of immune cells in adipose tissue [52]. Furthermore, this may suggest a role for adipose-derived ceramide in chronic and systemic inflammation via adipokine regulation in response to HFD.

In addition to adipokine regulation, ceramide has been shown to function in the regulation of adipose tissue. Inhibition of dihydroceramide desaturase reduced ceramide formation and increased dihydroceramide accumulation in 3T3-L1 mouse derived adipocytes and white adipose tissues in mice, leading to impaired differentiation and lipid accumulation [77]. This study implicates ceramide and dihydroceramide regulation of adipose tissue homeostasis.

C1P, generated by ceramide kinase, has not been well studied in response to HFD. The study that has been performed demonstrated that deletion of ceramide kinase abrogated increased mRNA expression of adipokines IL-6, TNF*α*, and MCP-1 and decreased macrophage recruitment to adipose tissue, in response to HFD [78]. This study suggests that ceramide kinase, and perhaps C1P, may function similarly to ceramide in the generation of adipokines.

S1PR modulators are being extensively utilized to define S1PR-mediated signaling events and pathobiologies [2]. Upon phosphorylation by SK2, FTY720 is able to bind S1PR1 inducing internalization and degradation of the receptor and sequestration of lymphocytes in secondary lymphoid tissues [79, 80]. In response to HFD, FTY720 prevented HFD-induced lymphocyte and macrophage infiltration in adipose tissue (although the mechanism for macrophages is unclear), resulting in reduced local inflammation and improved insulin resistance [81]. In addition to immune cell regulation, FTY720 has been shown to directly mediate HFD-induced effects on adipocytes. Phospho-FTY720 inhibited preadipocyte differentiation into mature adipocytes and stimulated lipolysis, reducing fat mass in HFD-fed mice [82]. In addition to receptor mediated effects on adipocytes, S1P itself has been implicated downstream of SK1 in lipid accumulation. Pharmacological or genetic inhibition of SK1 attenuated lipid accumulation in differentiating 3T3-L1 adipocytes [83]. These studies suggest involvement of S1P-S1PR signaling in HFD-induced adipose tissue dysregulation.

Sphingolipids in adipose tissue are potent mediators for adipokine expression and secretion in response to HFD. Moreover, sphingolipid enzymes and their product lipids may also regulate subsequent immune cell infiltration and proinflammatory signaling in adipose tissues in HFD and obesity.

### 4.4. Cardiovascular System

Systemic insulin resistance and excessive cytokine release due to obesity significantly alter the cardiovascular environment including blood pressure, coagulation, and fibrinolysis, ultimately leading to endothelial dysfunction and atherosclerosis [84]. Endothelial dysfunction is the failed balance between endothelium-dependent vasodilation and contraction. In addition to this imbalance, endothelial activation confers susceptibility to atherosclerosis [85]. Sphingolipids are significantly altered by HFD and palmitate treatment in the cardiovascular system, and these altered sphingolipids, specifically ceramide, seem to be essential in the regulation of obesity-induced cardiovascular diseases [86–89].

Endothelial dysfunction is mainly caused by dysregulation of endothelial nitric oxide synthase (eNOS), the enzyme that generates the potent vasodilator nitric oxide (NO) [85]. Palmitate- and HFD-induced generation of ceramide resulted in endothelial dysfunction, abolishing vascular endothelial growth factor (VEGF) or insulin-stimulated eNOS activation and subsequent NO production from endothelial cells [88, 89]. Inhibition of* de novo* synthesis with myriocin and SPTLC2 haploinsufficiency restored HFD-induced eNOS phosphorylation and achieved the normal range of vasodilation [88]. In addition, palmitate impaired angiogenesis in human umbilical vein endothelial cells (HUVECs) upon VEGF treatment, resulting in reduced tube formation in matrigel. This reduction in angiogenesis was rescued by myriocin, demonstrating the involvement of* de novo* generated sphingolipids in VEGF function [89].

Plasma sphingomyelin levels are reported to correlate with the increased risk of coronary heart disease [32]. In addition, high levels of sphingomyelin in low density lipoprotein (LDL) contributed to aggregation and retention of LDL in arterial wall. High levels of LDL-sphingomyelin supplied sufficient substrate for arterial SMase to increase LDL-ceramide, conferring aggravated atherosclerotic damage [90]. Consistently, LDL from SMS2 transgenic mice exhibit proatherogenic properties with increased* in vitro* aggregation upon SMase treatment [35]. Also, SMS2 overexpression in mice with adenovirus exaggerated atherosclerotic inflammation with elevations in cyclooxygenase-2 (COX-2) and matrix metalloproteinase-2 (MMP-2) in aorta [91]. Myriocin treatment decreased sphingomyelin and ceramide levels in plasma and consequently resulted in less atherosclerotic lesions in aorta from HFD-fed Apo-E deficient mice [87, 92, 93]. Moreover, bone marrow-derived SPTLC2 haploinsufficient macrophages also reduced atherosclerotic lesions in mice suggesting the importance of immune cell-derived sphingolipids in this disease (similar to adipose tissue) [86]. Meanwhile, the effect of glycosphingolipids and their metabolizing enzymes in atherosclerosis has been controversial [94–97]. The studies that have been conducted suggest that glycosphingolipids are necessary in atherosclerosis progress, but not a sufficient therapeutic target for this disease.

Altered sphingolipid metabolism has also been implicated in cardiomyopathy. A single study suggested that palmitate-induced ceramide and sphingomyelin accumulation in cardiac tissue resulted in Ca^++^ dysregulation and consequent systolic contractile dysfunction [98]. In a recent study by Russo et al., cardiac tissue was found to be enriched in SPTLC3, which has been shown to utilize the C14:0 saturated fatty acid myristate. This results in the* de novo* generation of d16:0/d16:1-base sphingolipids. Elevation of these d16:0/d16:1 sphingolipids resulted in increased apoptosis [25]. This study suggested the involvement of novel sphingolipid species in obesity-induced cardiomyopathy.

HFD-mediated dysregulation of sphingolipids contributes to obesity-related cardiovascular disease. In addition, ceramide and sphingomyelin contribute to endothelial dysfunction, atherosclerosis, and cardiomyopathy in cardiovascular tissues. Together these studies suggest that dietary fat overload and obesity alter cardiovascular function are at least partially due to altered sphingolipid metabolism.

## 5. Conclusion

Sphingolipid metabolism is significantly affected by dietary nutrient oversupply, including HFD. Due to the complexity of the sphingolipid metabolism network, it is hard to define the regulation and role of a single sphingolipid species in HFD-involved biologies and pathobiologies. However, HFD significantly alters numerous sphingolipids species, impacting the downstream sphingolipid-mediated cellular signaling pathways. These changes in cellular signaling often contribute to HFD-induced toxicity in affected tissues: liver, muscle, adipose, and cardiovascular. Therefore, dissecting HFD-mediated sphingolipid metabolism and understanding the mechanisms by which HFD regulates these changes may lend insight into potential therapeutic and nutritional targets in obesity-related diseases.

## Figures and Tables

**Table 1:** Ceramide regulation by HFD or fatty acids.

Tissue	Experimental model	Ceramide species	Alteration	Reference
Liver	60% HFD for 5 weeks in Wistar rats	Total ceramide	Increased	[40]
	60% HFD for 16 weeks in C57BL/6 mice	C14:0, C16:0, C18:0, C20:0, and	Increased	[9]
		C24:0	Decreased	
	30% HFD with 40% fructose for 2 weeks in Syrian Golden hamsters	C14:0, C18:0, C18:1, and C20:0	Increased	[39]
	34% HFD for 3 weeks in Wistar rats	C14:0, C16:0, C18:0, C18:1, and C24:1	Increased (nuclei)	[29]
		C14:0, C16:0, C18:0,	Increased (total)	
	42% HFD for 16 weeks in C57BL/6 mice	C18:0, and C20:0	Increased	[11]
		C24:0, C24:1	Decreased	
	42% HFD for 3 weeks in C57BL/6 mice	C20:0, C22:0	Increased	[11]
		C24:0, C24:1	Decreased	
	42% HFD for 6 weeks in C57BL/6 mice	C18:0, C20:0, C22:0, C24:1, C24:0, and total ceramide	Increased	[8]
	60% HFD for 4 weeks in C57BL/6 mice	C16:0, C22:0, and total ceramide	Increased	[12]
	0.3 mM palmitate for ≤24 h in primary rat hepatocytes	C16:0	Increased	[43]

Skeletal muscle	42% HFD for 16 weeks in C57BL/6 mice	C16:0, C18:0	Increased	[11]
	42% HFD for 3 weeks in C57BL/6 mice	C18:0	Increased	[11]
	42% HFD for 6 weeks in C57BL/6 mice	C16:0, C18:0, C20:0, C22:0, C24:1, C24:0, and total ceramide	Increased	[8]
	10-week HFD in 25-month-old Wistar rat	Total ceramide	Increased	[21]
	300 *μ*M palmitate for 3 days during differentiation in human myotubes	Total ceramide, primarily C16:0, but except for C24:1	Increased	[18]
	Obese patient samples	C16:0, C18:0, C20:0, C22:0, and total ceramide	Increased	[10]
	60% HFD for 6 weeks in C57BL/6 mice	C16:0, C18:0, C18:1, and total ceramide	Increased	[41]
	60% HFD for 10 weeks in C57BL/6 mice	C16:0, C18:0, C18:1, and total ceramide	Increased	[13]
	42% HFD for 6 weeks in C57BL/6 mice	C16:0, C18:0, and total ceramide	Increased	[68]
		C24:1	Decreased	
	0.75 mM palmitate for 16 h in C2C12 myotubes	C16:0, C22:0, and total ceramide	Increased	[12]
	60% HFD for 4 weeks in C57BL/6 mice	C16:0, C18:0, and total ceramide	Increased	[12]
	0.75 mM palmitate for 16 h in C2C12 myotubes	Total ceramide, total glucosylceramide	Increased	[65]
	0.75 mM palmitate for 16 h in C2C12 myotubes	Total ceramide	Increased	[23]
	1.25 mM palmitate for 14 h in C2C12 myotubes	C16:0, C18:0, C24:0, C24:1, and total ceramide	Increased	[14]

Adipose tissue	42% HFD for 16 weeks in C57BL/6 mice	C16:0, C18:0, C20:0, and C22:0	Increased	[11]
	Obese patient samples	C14:0, C16:0, C18:0, C18:1, and C22:1	Increased	[17]
	55% HFD for 18 weeks in C57BL/6 mice	C16:0, C18:0	Increased	[17]
		C22:0, C24:0	Decreased	
	60% HFD for 16 weeks in C57BL/6 mice	C16:0, C18:0, and C18:1	Increased	[16]
		C16:0, C18:0, and C18:1	Increased	

Plasma/serum	60% HFD for 16 weeks in C57BL/6 mice	C16:0, C18:0, C20:0, and C24:1	Increased	[16]
	31% HFD in diabetic Rhesus Macaque monkeys	C14:0, C16:0, C18:0, C20:0, C22:0, and C24:0	Increased	[7]
	60% HFD for 13 weeks in C57BL/6 mice	C20:0, C22:0, C24:0, and total ceramide	Increased	[30]
	60% HFD for 8 weeks in Long Evans rats	Total ceramide	Increased	[24]

Aorta	100 *μ*M palmitate for 8 h in primary bovine aortic endothelial cells	C16:0	Increased	[89]
	60% HFD for 2 weeks in C57BL/6 mice	C16:0, C18:0, and C22:0	Increased	[89]

Heart	45% HFD for 16 weeks in 18-month-old C57BL/6 mice	Total ceramide	Increased	[19]
	41% palmitate-enriched HFD for 12 weeks in C57BL/6	C16:0, C20:0, and C20:1	Increased	[98]
	60% HFD for 12 weeks in 40–44-week-old C57BL/6 mice	C18:0	Increased	[20]
	60% milk-fat based HFD for 8 weeks in C57BL/6 mice	d16:1-base ceramide	Increased	[25]
	60% HFD for 16 weeks in C57BL/6 mice	C16:0, C18:0, C18:1, C20:0, and C24:1	Increased	[15]

**Table 2:** Sphingomyelin regulation by HFD or fatty acids.

Tissue	Experimental model	Sphingomyelin species	Alteration	Reference
Liver	60% HFD for 5 weeks in Wistar rats	NS	Increased	[40]
	34% HFD for 3 weeks in Wistar rats	SM16:0, SM18:0, and SM18:1	Increased (nuclei)	[29]
		SM16:0, SM18:0	Increased (total)	
	58% HFD for 16 weeks in C57BL/6 mice	NS	Increased	[54]

Skeletal muscle	60% HFD for 6 weeks in C57BL/6 mice	SM18:1	Increased	[41]
		SM18:0, total SM	Decreased	

Adipose tissue	42% HFD for 16 weeks in C57BL/6 mice	SM14:0, SM16:0, SM16:1 SM18:0, and SM18:1	Increased	[11]

Plasma/serum	45% HFD for 14 weeks in C57BL/6 mice	SM16:0, SM18:0, SM16:1, SM18:1, and SM22:1	Increased	[33]
	58% HFD for 16 weeks in C57BL/6 mice	NS	Increased	[54]

Heart	60% HFD for 16 weeks in C57BL/6 mice	SM 16:0, SM18:0, and SM18:1	Increased	[15]
	41% palmitate-enriched HFD for 12 weeks in C57BL/6	SM16:0, SM20:0, SM24:0, and SM24:1	Increased	[98]

NS = not specified.

**Table 3:** Sphingosine and S1P regulation by HFD or fatty acids.

Tissue	Experimental model	Species	Alteration	Reference
Liver	0.3 mM palmitate for ≤24 h in primary rat hepatocyte	S1P	Increased	[43]
	30% HFD with 40% fructose for 2 weeks in Syrian Golden hamsters	Sphingosine	Increased	[39]
		S1P	No Change	
	60% HFD for 5 weeks in Wistar rats	Sphingosine, S1P	No Change	[40]
	58% HFD for 16 weeks in C57BL/6 mice	Sphingosine	Increased	[54]

Skeletal Muscle	60% HFD for 6 weeks in C57BL/6 mice	Sphingosine, S1P	No Change	[41]
	0.75 mM palmitate for 16 h in C2C12 cells	S1P	Increased	[65]
	1.25 mM palmitate for 14 h in C2C12 cells	S1P	Increased	[14]
		Sphingosine	No Change	
	0.75 mM palmitate for 16 h in primary mouse myotube	S1P	Increased	[48]

Adipose tissue	58% HFD for 16 weeks in C57BL/6 mice	Sphingosine	Increased	[54]

Plasma	42% HFD for 6 weeks in C57BL/6 mice	S1P	Increased	[42]
	42% HFD for 6 weeks in C57BL/6 mice	S1P	Increased	[8]
	58% HFD for 16 weeks in C57BL/6 mice	S1P	Increased	[54]
		Sphingosine	Increased	
